# Technical implementation for african pharmacogenetic studies on *CYP2D6* from archived breast cancer formalin fixed paraffin-embedded (FFPE) tissues

**DOI:** 10.1016/j.mex.2026.103946

**Published:** 2026-05-07

**Authors:** Keneuoe Cecilia Nthontho, Leabaneng Tawe, Nduta Mugo, Rorisang Mabaka, Gianmarco Contino, Andrew Khulekani Ndlovu, Daniel Paul Morse, Giacomo Maria Paganotti

**Affiliations:** a School of Allied Health Professions, Faculty of Health Sciences, University of Botswana, Gaborone, Botswana; b Botswana-University of Pennsylvania Partnership, Gaborone, Botswana; c Department of Cancer and Genomic Sciences, College of Medicine and Health, University of Birmingham, Edgbaston, Birmingham, B15 2TT, UK; d Department of Biomedical Sciences, Faculty of Medicine, University of Botswana, Gaborone, Botswana; e Department of Chemistry, US Naval Academy, Annapolis, MD 21402, USA; f Division of Infectious Diseases, Perelman School of Medicine, University of Pennsylvania, Philadelphia, PA 19104, USA

**Keywords:** ARMS-PCR, Breast cancer, *CYP2D6*, Formalin-fixed paraffin-embedded tissues, Nested-PCR, Pharmacogenetics, RFLP

## Abstract

Breast cancer remains the most common cancer affecting women globally, with a disproportionate impact on low- and middle-income countries (LMICs), where survival is poor despite lower incidence. Conducting molecular studies in these settings is often hampered by limited resources and technical challenges, particularly when using traditional DNA sources. This is also true for pharmacogenetic studies among Africans. For this, we propose novel *CYP2D6* PCR protocols optimized for formalin-fixed paraffin-embedded (FFPE) tissue blocks, a readily available and accessible resource in LMICs. Although formalin fixation can cause DNA fragmentation, FFPE tissues offer a valuable source for genetic studies.

• We introduce four nested PCR-RFLP approaches targeting *CYP2D6* variants for *2, *4, *17, *29 alleles (rs16947, rs3892097, rs28371706, rs59421388), which influence drug metabolism, in particular tamoxifen (breast cancer treatment)
• Additionally, two amplification-refractory mutation system (ARMS) protocols are proposed to detect variants that determine CYP2D6*2 (rs5758550) and CYP2D6*29 (rs61736512) that lack suitable cleavage sites.

This workflow aims to demonstrate the feasibility of pharmacogenetic studies in LMICs using FFPE samples, providing alternative techniques to traditional methods. Understanding *CYP2D6* genetic variations is crucial in African populations, where tamoxifen metabolism impacts treatment efficacy. This research could improve personalized therapy and reduce mortality disparities in sub-Saharan Africa.

## Graphical abstract

**Figure:**
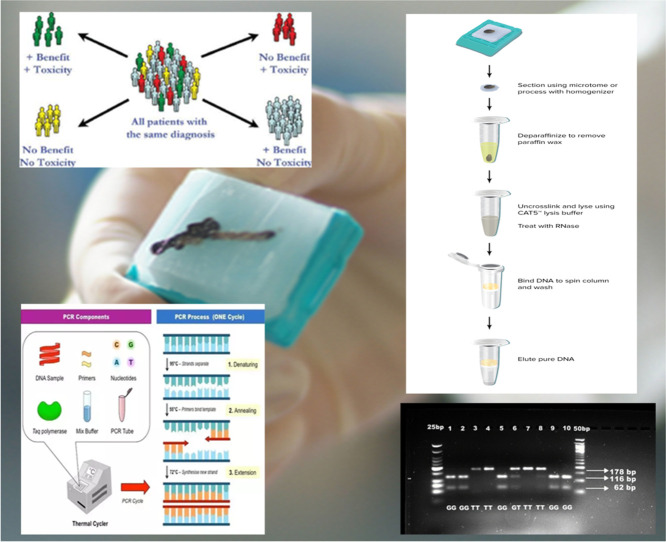

## Specifications table

This table provides general information on your method.

**Table:** 

**Subject area**	Technical protocol, Genetics and Molecular Biology
**More specific subject area**	Pharmacogenetics
**Name of your method**	CYP2D6 genotyping for African genetic variants from FFPE
**Name and reference of original method**	Quick-DNA FFPE Miniprep (Zymo Research, Irvine, CA, USA) method modification, new list of primers for different PCR applications, and RFLP (Naveen et al., 2006; Bright et al., 2013) and ARMS analysis
**Resource availability**	https://files.zymoresearch.com/protocols/_d3067_quick-dna_ffpe_miniprep.pdf Table 1 and 2, Figures 1-6, Supplementary Material

## Background

Breast cancer is the most common cancer affecting women worldwide, with approximately two thirds of related deaths occurring in low- and middle-income countries (LMICs), including Africa [1]. Though the incidence of breast cancer appears to be relatively low in Africa, survival from the disease is markedly poor in the region, with high mortality recorded in many settings [2]. Limited resources and technical constraints may hamper analysis of specimen (biopsies, blood, etc.) aimed to detect, genotype and characterize breast cancer in LMICs. Quantitative approaches to PCR are sometimes non-available, as well as the source of DNA can provide low quality nucleic acid extracts. For these reasons, we are proposing and validating new PCR protocols modified for formalin-fixed paraffin embedded (FFPE) tissue blocks. Retrospective studies on both germline and/or somatic DNA are often conducted using biological material preserved into the FFPE blocks. From one side, FFPE blocks could be considered a relatively good (and easy to obtain, store and preserve) source of DNA for molecular studies [3,4], and using these as source of DNA dramatically increases the availability of cohorts for prospective–retrospective studies [5]. However, formalin fixation may cause extensive cross-linking and fragmentation [3,6] resulting in significant damage to the viable DNA material [7]. In addition, genetic aberrations, and/or insufficient DNA extraction should be considered when working with nucleic acid extraction from FFPE material. Even so, in LMICs laboratories, FFPE blocks are largely used for molecular studies when other DNA sources are unavailable. Here we describe four new nested PCR- Restriction Fragment Length Polymorphism (PCR-RFLP) assays for CYP2D6*2 (rs16947), CYP2D6*4 (rs3892097), CYP2D6*17 (rs28371706), CYP2D6*29 (rs59421388). Because some polymorphisms lack suitable restriction sites, we additionally developed an amplification-refractory mutation system (ARMS) to enable discrimination of CYP2D6*2 (rs5758550) and CYP2D6*29 (rs61736512) [8].

With the proposed workflow, our aim is to evaluate the feasibility of applying pharmacogenetic analysis to nucleic acids extracted from FFPE. The field of pharmacogenetics is rapidly expanding, and its clinical implementation has become increasingly feasible with the proliferation of pharmacogenetic tests. These tests are important in highlighting the genetic interplay between drug metabolism and how individuals respond differently to certain drug treatments.

The need for accessible pharmacogenetic testing is particularly acute in LMICs. In sub-Saharan Africa, breast cancer incidence remains relatively low, yet mortality is disproportionately high. Variation in drug-metabolizing enzymes may contribute to these outcomes. This is especially relevant for tamoxifen, widely used for estrogen receptor–positive (ER+) breast cancer, which requires hepatic bioactivation by *CYP2D6* to form endoxifen. *CYP2D6* is highly polymorphic, and the distribution of its alleles varies markedly across populations. In this study, we focus on alleles commonly found in African populations, specifically CYP2D6*2, *4, *17 and *29 [9].

## Method details

### DNA extraction

Firstly, we introduced several protocol modifications to the DNA extraction method provided by Zymo Research DNA extraction kits (https://files.zymoresearch.com/protocols/_d3067_quick-dna_ffpe_miniprep.pdf).

1. Tissue sectioning

Instead of up to four (4) tissue sections ≤20 µm thick, we optimized the protocol on **five (5) sections with a thickness of 10 μm each**. This approach enhances tissue exposure and improves penetration of the deparaffinizing solution.

2. Pre-dewaxing

We recommend a **pre-dewaxing step** in which 1 ml of xylene or other commercial xylene substitutes (e.g. Histo-Clear) is added to the tissue and incubated overnight, to ensure complete removal of the paraffin wax.

3. Deparaffinization (dewaxing)

After adding 400 μl of Deparaffinization Solution (as suggested by the manufacturer’s protocol), we introduce a pre-step of **vigorous vortexing of 10 seconds, followed by a brief centrifugation in order to bring the** material **to the bottom of the tube**. The samples are then incubated at 55°C for **3 minutes instead of 1 minute** (as recommended by the manufacturer) and finally **allowed to cool to room temperature**.

4. Tissue Digestion

During the tissue digestion step, 45 μl of DNase/RNase-free water, 45 μl of 2X Digestion Buffer and 10 μl of Proteinase K should be added to the sample (as suggested by the manufacturer’s protocol). **We suggest vortex and briefly centrifuge the sample in order to pellet the debris. Then, instead of a 1- 4-hour incubation time at 55°C, we suggest adopting the option of “Standard Digestion” overnight**.

Total nucleic acid purification in the final purification step, after adding the Elution Buffer to the columns**, we recommend closing the lid and incubating at room temperature (15–25°C) for 30 minutes, instead of 2-5 minutes suggested by the manufacture**. As the final step, we recommend **centrifuging at maximum speed for 10 minutes** instead of 30 seconds to maximize DNA yield, providing an optimal template for subsequent PCR.

### Genotyping

We designed several primers for PCR approaches that sharply increase PCR efficiency. The improved methods are based on nested PCR-RFLP and nested ARMS protocols. For the nested PCR-RFLP primers, amplicon sizes and restriction fragments are illustrated in Table 1. Nested PCR and RFLP analysis are validated and widespread approaches also used for pathogen detection (e.g. malaria and other microbes) and other molecular applications, including genotyping [10].

**Table 1:** Oligonucleotides specifications, amplicon sizes, restriction enzymes and fragment size for nested PCR-RFLP.

*CYP2D6* SNPs	Outer primers	PCR size [and reference]		Nested primers	PCR size [and reference]	RFLP
*2 (rs16947) 2850 C>T R296C	GGTTGGACCAGTGCATCA	360 bp [*in house*]		GGCTGACAGGTGCAGAA	245 bp [*in house*]	*Hha1* 125 bp and 120 bp (cuts the wild-type C allele)
	CTGGTCAAGCCTGTGCT			CCTGCACTGTTTCCCAGATG		
*4 (rs3892097) 1846 G>A Splicing Defect/Null	CAAGAAGTCGCTGGAGCAGT	255 bp [13]		TGGGTGATGGGCAGAAGG	155 bp [*in house*]	*BstN1* 69 bp and 86 bp (cuts the wild-type G allele)
	GAGGGTCGTCGTACTCGAAG			GATCGCCTCCCTCACCTG		
*17 (rs28371706) 1023 C>T T107I	GGACTAGGACCTGTAGTCTG	[*in house*]	280 bp	GGACGTGTTCAGCCTGCA	165 bp [*in house*]	*Fok1* 107 bp and 58 bp (cuts the variant T allele)
	CCCGGGTCCCACGGAAATCT	[14]		CGCTGCTTGCCTTGGGAA		
*29 (rs59421388) 3184 G>A V338M	CCATCTGGGAAACAGTGCA	310 bp [*in house*]		GCCTGACCTCCTCCAACATA	178 bp [*in house*]	*HpyCh4IV* 116 bp and 62 bp (cuts the wild-type G allele)
	CGCTGCACCTCATGAATCA			CAGTGGTGTAGGGCATGTGA		

In addition, we designed nested ARMS primers (Table 2). These two nested ARMS protocols enable genotyping of two “African” *CYP2D6* variants where a restriction enzyme cannot cleave the polymorphic site. Briefly, an outer-PCR will provide the template for the ARMS. A single PCR master mix is then prepared with all four (4) primers. This technique selectively amplifies a target allele, either the wild-type and/or the mutant allele. The principle relies on designing primers with a mismatch at the 3′ end, preventing successful extension and amplification of the non-complementary allele [11,12].

**Table 2:** Oligonucleotides specifications and amplicon sizes for the nested ARMS PCR. Mismatched nucleotides are in bold.

*CYP2D6* SNPs	Outer primers	ARMS primers	ARMS-PCR size
*2 (rs5758550) 457 A>G Enhancer Variant	AGCTTACTTACTGAGATTTGGCT (forward)	CTTAAGCATTCCCCTAGTTACCATAAGC (control forward)	Product size of two control primers: 313 bp Product size for G allele: 171 bp Product size for A allele: 197 bp
		TCAGATTTCTCAACAGGAAAGTCACATA (control reverse)	
	TGATAGGCAAGGAGAAACTGTG (reverse)	AAAAGACTATGAAAATACAGGACATT**C**GC (G allele)	
		AAAGATTCCCATTCCACAGTTTT**G**TA (A allele)	
*29 (rs61736512) 1660 G>A V136I	GACAGATTTCCGTGGGACCC (forward)	ATAGGGTTGGAGTGGGTGGTGGATG (control forward)	Product size of two control primers: 307 bp Product size for A allele: 151 bp Product size for G allele: 200 bp
		GGAGATGCGGGTAAGGGGTCG (control reverse)	
	GAAAGCCCGACTCCTCCTTC (reverse)	CCCAAGTTGCGCAAGGTGGA**A**AT (A allele)	
		GCGAGCAGAGGCGCTTCT**A**CG (G allele)	

Of relevance, all the proposed protocols may work efficiently on “normal” non-FFPE extracted DNA, just excluding the first amplification step (outer PCR) for all the new protocols in this paper.

For all PCR experiments we used 4 µl DNA template (see DNA extraction section), in the first reaction (outer PCR) in a total volume of 20 µl (see Table 1, Table 2).

(1) For the nested RFLP PCR, we used 2 µl of the outer PCR product as template for the nested reaction. Below is a list of specific conditions for PCR, by SNP:
a) CYP2D6*2 (rs16947), 2850 C>T, R296C

**Fig. 1:**
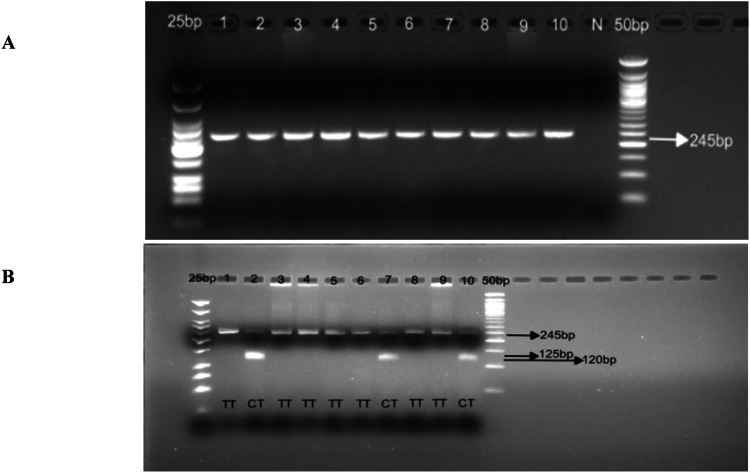
Gel pictures showing nested PCR (A) and RFLP analysis (B) for CYP2D6*2 rs16947. Line 1-10 (A) nested PCR amplicons; N: negative control. PCR products were run on 2% agarose gel. Line 1-10 (B) *Hha1* digestion of the nested PCR. Digestion products were run on 3% agarose gel.

[MgCl_2_] = 2.5mM for both outer and nested PCR; annealing temperature (Ta) for outer PCR = 52°C (35 cycles); Ta for nested PCR = 57°C (40 cycles). See Fig. 1

b) CYP2D6*4 (rs3892097), 1846 G>A, splicing defect/null

**Fig. 2:**
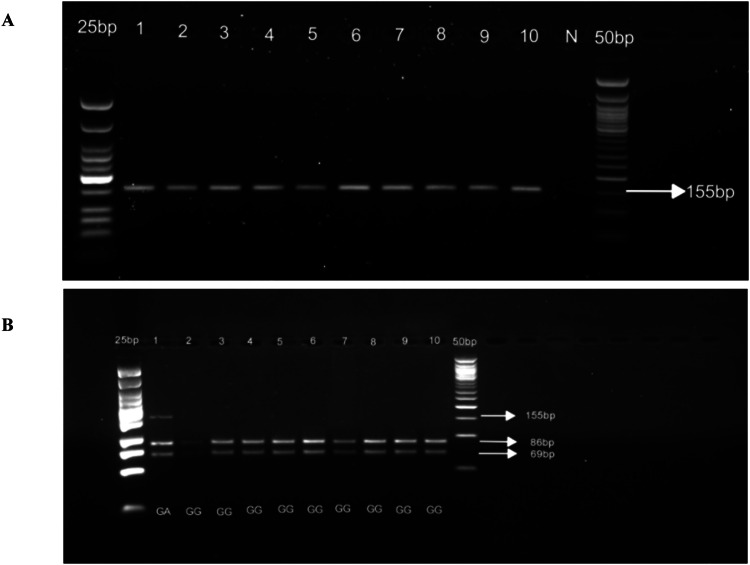
Gel pictures showing nested PCR (A) and RFLP analysis (B) for CYP2D6*4 rs3892097. Line 1-10 (A) nested PCR amplicons; N: negative control. PCR products were run on 2% agarose gel. Line 1-10 (B) *BstN1* digestion of the nested PCR. Digestion products were run on 3% agarose gel.

[MgCl_2_] = 2.5mM for both outer and nested PCR; Ta for outer PCR = 60°C (40 cycles); Ta for nested PCR = 64°C (35 cycles). See Fig. 2

c) CYP2D6*17 (rs28371706), 1023 C>T, T107I

**Fig. 3:**
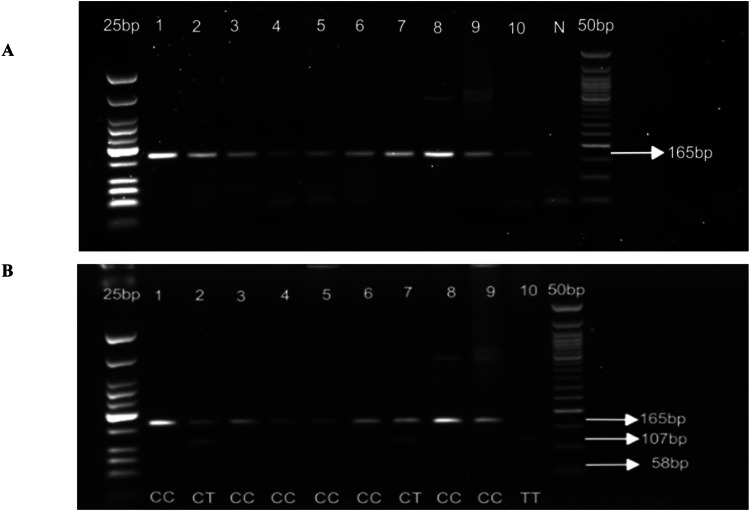
Gel pictures showing nested PCR (A) and RFLP analysis (B) for CYP2D6*17 rs28371706. Line 1-10 (A) nested PCR amplicons; N: negative control. PCR products were run on 2% agarose gel. Line 1-10 (B) *Fok1* digestion of the nested PCR. Digestion products were run on 3% agarose gel.

[MgCl_2_] = 3.5mM and [DMSO] = 5% of the PCR master mix volume (1 µl) for outer PCR; [MgCl_2_] = 2.5mM for nested PCR; Ta for outer PCR = 55°C (40 cycles); Ta for nested PCR = 60°C (35 cycles). See Fig. 3

d) CYP2D6*29 (rs59421388), 3184 G>A, V338M

**Fig. 4:**
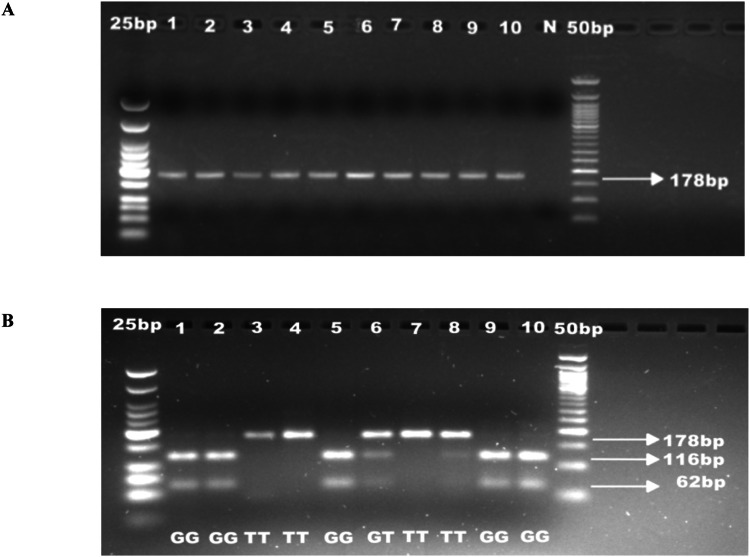
Gel pictures showing nested PCR (A) and RFLP analysis (B) for CYP2D6*29 rs59421388. Line 1-10 (A) nested PCR amplicons; N: negative control. PCR products were run on 2% agarose gel. Line 1-10 (B) *HpyCh4IV* digestion of the nested PCR. Digestion products were run on 3% agarose gel.

[MgCl_2_] = 3.5mM for both outer and nested PCR; Ta for outer PCR = 52°C (35 cycles); Ta for nested PCR = 55°C (40 cycles). See Fig. 4

(2) For the nested ARMS, a touchdown PCR approach was used for the outer PCR product in both reactions. Below is a list of specific conditions for the single PCR reaction, by SNP:
α) CYP2D6*2 (rs5758550), 457 A>G, enhancer variant

**Fig. 5:**
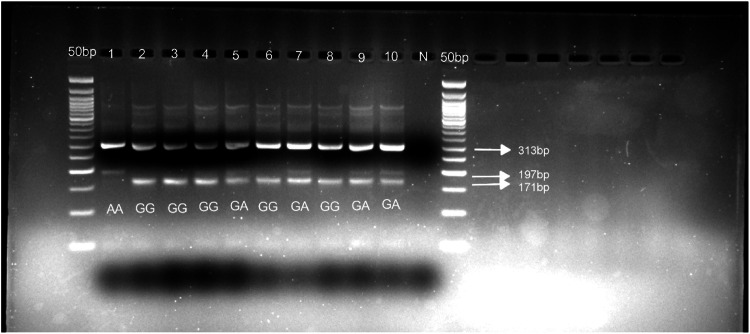
Gel picture showing ARMS PCR analysis for CYP2D6*2 rs5758550. Line 1-10 ARMS PCR amplicons; N: negative control. PCR products were run on 3% agarose gel.

[MgCl_2_] = 3mM for both outer and nested ARMS PCR; Ta for touchdown outer PCR = 55°C (10 cycles) with a 0.5°C temperature decrease down to 50°C (29 cycles); Ta for nested ARMS PCR = 64°C (25 cycles). See Fig. 5

β) CYP2D6*29 (rs61736512), 1660 G>A, V136I

[MgCl_2_] = 3mM for both outer and nested ARMS PCR; Ta for touchdown outer PCR = 55°C (10 cycles) with a 0.5°C temperature decrease down to 50°C (29 cycles); Ta for nested ARMS PCR = 64°C (25 cycles).

**Fig. 6:**
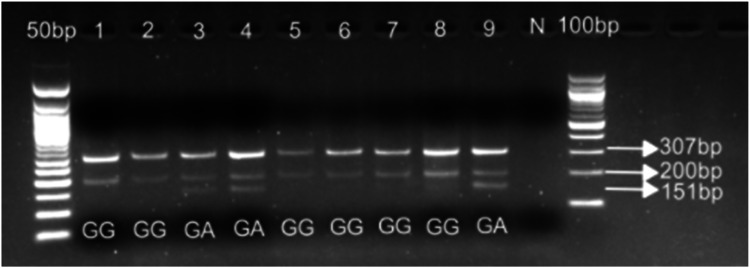
Gel picture showing ARMS PCR analysis for CYP2D6*29 rs61736512. N: negative control. PCR products were run on 3% agarose gel.

N.B. Due to a large PCR fragment (972bp) for rs61736512, we could not amplify the DNA from FFPE tissues despite countless optimization attempts. Amplification was, however, possible for samples that are not of FFPE origin. Technique non validated. See Fig. 6.

## Method validation

The proposed protocol modifications to the DNA extraction method (pre-dewaxing, step2) resulted in higher DNA concentration and purity. We tested ten samples with the two methods, and we achieved an average of 852.63 ng/ul and 2250.94 ng/ul for standard and modified protocols, respectively; with a 1.58 and 1.88 OD_260/280_ ratio.

The proposed protocols have been validated by Sanger sequencing of the PCR amplicons. See Supplementary materials. Specifically, we provide Sanger sequences for all the three genotypes (wild-type, heterozygous and double mutant, when available) by SNP. Sequencing work confirms the genotyping approaches proposed in this paper.

## Limitations

1. The highly fragmented DNA from FFPE blocks challenged the successful amplification of DNA from large fragment sizes, and in particular CYP2D6*29 (rs61736512), 1660 G>A, with a fragment size of 972bp for the outer PCR product, therefore we could not validate it through Sanger sequencing. Contrarily, DNA sourced from non-FFPE blocks was successfully amplified indicating that the protocol is effective under optimal DNA quality conditions.
2. Because of the origin of the patient samples (African ancestry), where we have noted a significantly low allele frequency [9], we were unable to find the mutant homozygous genotype in the sequences for CYP2D6*4 (rs3892097) and CYP2D6*29 (rs59421388). In addition, we were not able to find any wild-type genotype for CYP2D6*2 (rs5758550).

## Ethics statements

Ethical approval for using FFPE tissues from the National Health Laboratory in Gaborone, Botswana, was obtained from the Institutional Review Board (IRB) at the University of Botswana (Protocol # UBR/RES/IRB/BIO/208) and from the Ministry of Health and Wellness, Botswana (Protocol # HPDME: 13/18/1, 23^rd^ July 2020)

## CRediT author statement

**Keneuoe Cecilia Nthontho**: Conceptualization, Original draft preparation, Writing, Methodology. **Leabaneng Tawe**: Methodology, Writing, Formal analysis. **Nduta Victoria Mugo**: Methodology, Data validation. **Rorisang Mabaka**: Data validation, Data curation. **Gianmarco Contino**: Data validation, Writing, Review and editing. **Andrew Khulekani Ndlovu**: Supervision, Resources, Writing, Review and editing. **Daniel Paul Morse**: Conceptualization, Methodology, Data curation. **Giacomo Maria Paganotti**: Conceptualization, Writing, Review and editing.

## Declaration of competing interest

The authors declare that they have no known competing financial interests or personal relationships that could have appeared to influence the work reported in this paper.

## Data Availability

Data will be made available on request.

## Appendix Supplementary materials

Image, application 1

## References

[1] Iqbal J. (2025). Women-Centric Breast Cancer Care in Low- and Middle-Income Countries: Challenges, Solutions, and a Roadmap for Equity. Cancer Control.

[2] Sung H., Ferlay J., Siegel R.L., Laversanne M., Soerjomataram I., Jemal A., Bray F. (2021). Global Cancer Statistics 2020: GLOBOCAN estimates of incidence and mortality worldwide for 36 cancers in 185 countries. CA Cancer J. Clin..

[3] Gaffney E.F., Riegman P.H, Grizzle W.E., Watson P.H (2018). Factors that drive the increasing use of FFPE tissue in basic and translational cancer research. Biotech. Histochem..

[4] Wilkins A., Chauhan R., Rust A., Pearson A., Daley F., Manodoro F., Fenwick K., Bliss J., Yarnold J., Somaiah N. (2018). FFPE breast tumour blocks provide reliable sources of both germline and malignant DNA for investigation of genetic determinants of individual tumour responses to treatment. Breast Cancer Res. Treat..

[5] Hertz D.L., Kidwell K.M., Thibert J.N., Gersch C., Regan M.M., Skaar T.C., Henry N.L., Hayes D.F., Van Poznak C.H., Rae J.M. (2015). Genotyping concordance in DNA extracted from formalin-fixed paraffin embedded (FFPE) breast tumor and whole blood for pharmacogenetic analyses. Mol. Oncol..

[6] Tawe L., Grover S., Narasimhamurthy M., Moyo S., Gaseitsiwe S., Kasvosve I., Paganotti G.M. (2018). Molecular detection of human papillomavirus (HPV) in highly fragmented DNA from cervical cancer biopsies using double-nested PCR. MethodsX.

[7] Oba U., Kohashi K., Sangatsuda Y., Oda Y., Sonoda K.H., Ohga S., Yoshimoto K., Arai Y., Yachida S., Shibata T., Ito T., Miura F. (2022). An efficient procedure for the recovery of DNA from formalin-fixed paraffin-embedded tissue sections. Biol. Methods Protoc..

[8] Ye S., Dhillon S., Ke X., Collins A.R., Day I.N (2001). An efficient procedure for genotyping single nucleotide polymorphisms. Nucleic Acids Res..

[9] Nthontho K.C., Ndlovu A.K., Sharma K., Kasvosve I., Hertz D.L., Paganotti G.M. (2022). Pharmacogenetics of Breast Cancer Treatments: A Sub-Saharan Africa Perspective. Pharmgenomics Pers. Med..

[10] Green M.R., Sambrook J. (2019). Nested Polymerase Chain Reaction (PCR). Cold Spring Harb. Protoc..

[11] Little S. (2001). Amplification-refractory mutation system (ARMS) analysis of point mutations. Curr. Protoc. Hum. Genet. Chapter.

[12] Medrano R.F., de Oliveira C.A. (2014). Guidelines for the tetra-primer ARMS-PCR technique development. Mol. Biotechnol..

[13] Bright A.T., Alenazi T., Shokoples S., Tarning J., Paganotti G.M., White N.J., Houston S., Winzeler E.A, Yanow S.K (2013). Genetic analysis of primaquine tolerance in a patient with relapsing vivax malaria. Emerg. Infect. Dis..

[14] Naveen A.T., Adithan C., Soya S.S., Gerard N., Krishnamoorthy R. (2006). CYP2D6 genetic polymorphism in South Indian populations. Biol. Pharm. Bull..

