# Supplementary material for: Technical implementation for african pharmacogenetic studies on CYP2D6 from archived breast cancer formalin fixed paraffin-embedded (FFPE) tissues
Source: MethodsX. 2026 May 7;16:103946. doi: 10.1016/j.mex.2026.103946 (PMC13191098; doi:10.1016/j.mex.2026.103946)
Supplement: Supplementary file 1 [file mmc1.docx]

**Supplementary Materials**: Sanger sequences

| ***CYP2D6*2*** | | | |
| --- | --- | --- | --- |
| **rs16947,** **2850 C>T** | | | |
|  | **CC** | **CT** | **TT** |
| **Forward** | 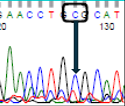 | 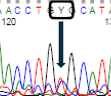 | 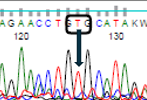 |
|  | | | |
|  | **GG** | **AG** | **AA** |
| **Reverse** | 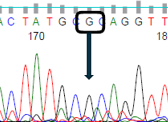 | 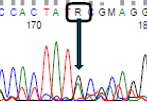 | 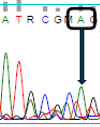 |
|  |  |  |  |

**Table 1**. *CYP2D6*2* variant (rs16947) showing both the forward and reverse genotypes.

| ***CYP2D6*2*** | | |
| --- | --- | --- |
| **rs5758550, 457 A>G** | | |
|  | **AG** | **GG** |
| **Forward** | **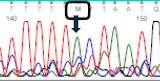** | **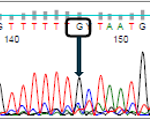** |
|  | | |
|  | **TC** | **CC** |
| **Reverse** | **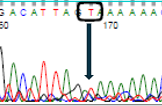** | **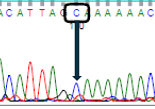** |
|  |  |  |

**Table 2**. *CYP2D6*2* variant (rs5758550) showing both the forward and reverse genotypes.

| ***CYP2D6*4*** | |  |
| --- | --- | --- |
| **rs3892097,1846 G>A** | |  |
|  | **GG** | **GA** |
| **Forward** | **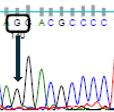** | **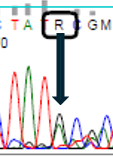** |
|  | |  |
|  | **CC** | **CT** |
| **Reverse** | **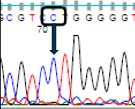** | **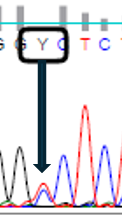** |
|  |  |  |

**Table 3**. *CYP2D6*4* variant (rs3892097) showing both the forward and reverse genotypes.

| ***CYP2D6*17*** | | | |
| --- | --- | --- | --- |
| **rs28371706, 1023 C>T** | | | |
|  | **CC** | **CT** | **TT** |
| **Forward** | **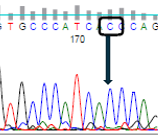** | **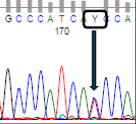** | **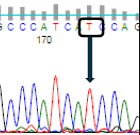** |
|  | | | |
|  | **GG** | **AG** | **AA** |
| **Reverse** | **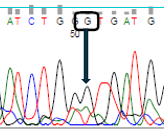** | **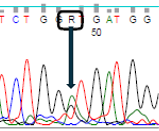** | **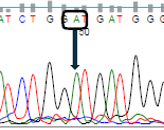** |

**Table 4**. *CYP2D6*17* variant (rs28371706) showing both the forward and reverse genotypes.

| ***CYP2D6*29*** | | |
| --- | --- | --- |
| **rs59421388, 3184 G>A** | | |
|  | **GG** | **GA** |
| **Forward** | **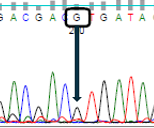** | **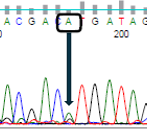** |
|  | | |
|  | **CC** | **CT** |
| **Reverse** | **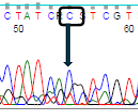** | **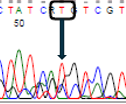** |

**Table 5**. *CYP2D6*29* variant (rs59421388) showing both the forward and reverse genotypes.
